# Teahouses as community third places: a grounded theory study of community-based health intervention for mental health promotion among urban older adults in China

**DOI:** 10.3389/fpubh.2026.1772758

**Published:** 2026-04-17

**Authors:** Weihua Tan, Jian Liu, Ruotong Zeng

**Affiliations:** 1 School of Politics and Public Administration, Hunan Normal University, Changsha, China; 2 Research Center for Chinese Ethical Civilization, Hunan Normal University, Changsha, China

**Keywords:** aging in place, China, emotional community, grounded theory, mental health, social isolation, social support, third place

## Abstract

**Objectives:**

The research aims to address social isolation and psychological distress among retired urban older adults in China amid rapid population aging and its challenges to public health and social care systems, explore the role of traditional urban teahouses as accessible “third places” in alleviating these issues, and investigate how these teahouses foster emotional communities for older adults.

**Methods:**

A qualitative grounded theory study was conducted at the Dongmao Street Teahouse in Changsha. Data were collected through participant observation and in-depth interviews with 25 older adults (aged 65+). The data were analyzed using a three-stage coding process (open, axial, and selective) to develop a theoretical model.

**Results:**

The analysis revealed a core process of emotional community formation, driven by a Nostalgic Spatial Setting. This setting facilitates Routinized Social Practices, enables Deep Emotional Interactions (including life-story sharing and public discourse), and encourages Cultural Identity Performance. These interconnected processes culminate in the generation of Emotional Belonging, characterized by place attachment, stress relief, and strong group identity, marking the establishment of a supportive emotional community. However, the sustainability of this community is challenged by the tension between maintaining authenticity for older patrons and adapting for cultural transmission to younger generations.

**Conclusion:**

Urban teahouses can function as vital community-based assets for healthy aging. They provide low-cost, accessible environments that facilitate meaningful social engagement, strengthen social support networks, and enhance the mental wellbeing of older adults through organic emotional community building. Public health and urban planning initiatives should recognize and support such grassroots, culturally embedded social spaces as components of age-friendly communities.

## 1 Introduction

Population aging has emerged as a defining feature of the global demographic transition, and mental health issues among older adults arising from social isolation and psychological distress have become a common challenge in the global public health field. Exploring culturally grounded and community-embedded approaches to mental health promotion for older adults has thus become a central agenda for active aging practices worldwide. China is experiencing a demographic shift towards a deeply aging society, with individuals aged 65 and over comprising 15.4% of the population (1). This transition poses profound public health and socio-economic challenges, including increased prevalence of age-related mental health issues such as depression and loneliness (2, 3). The national “active aging” strategy emphasizes community-based health promotion, promoting health, participation, and security to improve quality of life in old age (4–6). However, top-down approaches to older adult care and social participation often overlook the nuanced psychosocial needs of older adults, particularly their need for autonomous, meaningful social connection and emotional support after retirement (7, 8). As the developing country with the largest scale and the fastest pace of population aging, China’s exploration of culturally grounded, community-based intervention pathways for older adults’ mental health will serve as an important reference model for active aging practices within the global multicultural context.

Globally, third places—social environments distinct from home (the “first place”) and the workplace (the “second place”)—serve as core carriers of informal health promotion (9). For instance, cafes in Western Europe foster intergenerational connections, and community centers in America reduce isolation through structured activities (10). These studies highlight third places are cost-effective, stigma-free alternatives to clinical mental health interventions. Yet existing research still has notable theoretical limitations, and the integration of domestic indigenous research with global scholarship has also revealed deficiencies in localized analysis. Together, these constitute the core theoretical gaps in current research, which are reflected in two main aspects: First, research on third places and older adult health is plagued by dual limitations in cultural context and mechanistic exploration. Most extant studies are situated within the Western cultural context of individualism and commercialized operational models, failing to investigate the unique socio-emotional mechanisms of indigenous traditional spaces in non-Western collectivist cultures. Furthermore, such research merely scratches the surface of the correlational link between third places and social connectivity among older adults, failing to clarify the dynamic transformation process of “physical spaces facilitating mental health” and lacking in-depth qualitative analysis based on grounded theory.

Second, domestic research on the older adult in China has dual shortcomings in research focus and analytical depth. Relevant studies have mostly centered on formal older adult social spaces planned top-down by the government or communities, such as community activity centers and universities for the older adult, with insufficient attention paid to informal grassroots spaces like teahouses—where older adults gather spontaneously and which are deeply rooted in local history and regional culture. In addition, most studies only provide descriptive accounts of older adults’ spatial usage behaviors, without developing systematic theoretical frameworks for the health promotion mechanisms of such indigenous spaces. Nor have they examined the potential impacts of the sustainability tensions between preserving the authentic character of these spaces and advancing cultural inheritance on older adults’ psychological experiences and identity construction.

Communities serve as the pivotal setting for implementing health promotion interventions targeting older adults (11). The loss of occupational roles due to retirement, social restrictions imposed by public health emergencies, and the inadequate provision of age-friendly public spaces amid urban renewal are likely to result in the continuous atrophy of older adults’ social networks and a marked exacerbation of loneliness and social isolation (12–14). Although top-down constructed formal social spaces for older adults, such as community activity centers and universities for the older adult, have been established nationwide, their structured and standardized service models often fail to meet older adults’ demands for informal, culturally familiar, and self-directed social participation (15). Compounded by the fact that urban development tends to prioritize the construction of commercial and public facilities for younger groups, the accessibility of suitable social spaces for older adults is further diminished (16, 17). Therefore, identifying and supporting such organically formed, community-based indigenous spaces that are spontaneously used by older adults and can enhance their mental health has become a core imperative in the field of public health.

As a uniquely Chinese type of indigenous micro-public space, traditional urban teahouses are key venues where older adults gather spontaneously and develop a strong sense of place attachment (18). This study conceptualizes teahouses through the lens of Ray Oldenburg’s Third Place theory—these social environments, distinct from the home (the first place) and the workplace (the second place), feature the core characteristics of neutrality and accessibility, and act as carriers for informal social interaction (19). Accessible Third Places are critical for older adults to maintain social connectivity and are also widely recognized as a vital factor influencing their physical and mental health (20, 21). Teahouses not only align with the core attributes of Third Places but also possess a unique potential for nurturing emotional communities due to their deep embeddedness in collectivist culture, making them an important carrier linking physical spaces to the mental health of older adults. Against the backdrop of the aforementioned theoretical gaps, this study adopts the Grounded Theory approach and takes traditional urban teahouses as the research subject to propose three core research questions: First, within the context of Chinese cities, what are the dynamic construction process and inherent operational mechanisms of the emotional communities for older adults formed around traditional teahouses as Third Places? Second, what specific impacts do teahouse-based emotional communities exert on the mental health of urban older adults (e.g., alleviation of loneliness and psychological stress), and what are their underlying mechanisms of action? Third, what are the universally applicable core logics and context-specific indigenous elements of the teahouse model respectively? How can its transferable core experiences be translated into a reference for public health policies on active aging within the global multicultural context?

The Dongmao Street Teahouse in Changsha serves as the typical case for this study, whose representativeness fully aligns with the theoretical sampling principles of “typicality and information richness” in Grounded Theory. The core justifications are elaborated in the following three dimensions: First, the typicality of spatial attributes. Located in the old urban area of Changsha, the teahouse was renovated from a former factory building and fully preserves the regional cultural symbols of old Changsha and carriers of collective memory. It not only fits the core characteristics of the Third Place—neutrality, accessibility, and informality—but also distinguishes itself from commercial internet-famous tea shops and top-down government-led formal community activity spaces. Thus, it stands as a typical representative of indigenous traditional public spaces in China. Second, the typicality of the operational model. The teahouse adopts a market-oriented operation model with highly weakened commercial attributes. With an extremely low consumption threshold and open spatial management, it has formed a stable group of older adult regulars. The social order and interaction norms within the space are all spontaneously formed by older adult users through long-term daily interactions. The operator only undertakes basic service guarantee functions and does not interfere with the social interaction process of older adults, which fully embodies the “bottom-up” characteristic defined in this study: the formation and operation of the community are centered on the autonomous leadership and spontaneous interaction of older adults, rather than the top-down design and control by the government or operators. Third, the typicality of population characteristics. The older adult regulars of the teahouse are mainly retired older adults in Changsha’s urban areas, who make high-frequency visits weekly and have formed sustained and stable social interactions. This makes it an ideal setting for studying the construction of emotional communities among older adults in indigenous informal spaces. It functions not only as a commercial establishment but also as a repository of local culture and collective memory (22), providing a familiar and emotionally resonant scenic scaffold for social interaction. This study posits that such spaces can evolve beyond a mere “Third Place” into what we term an emotional community: a social network characterized by sustained emotional interaction, mutual support, shared identity, and a deep sense of belonging (23, 24). The formation of such communities represents a powerful bottom-up mechanism for combating loneliness and promoting psychosocial health among older adults.

While existing research has examined third places and community cohesion (25, 26), a gap remains in understanding the specific processes through which physical spaces like teahouses transform into psychological resources—emotional communities—for older adults within non-Western, rapidly aging urban contexts. This study takes the Dongmao Street Teahouse in Changsha as a typical case, exploring how teahouses nurture emotional communities for older adults through participant observation and in-depth interviews, and aims to make two core research contributions. Theoretically, it breaks the cultural contextual limitations of Western third place theory, extends its application in non-Western collectivist cultures, and constructs a dynamic formation model of older adult emotional communities driven by a nostalgic spatial setting. Practically, it systematically excavates the health promotion value of indigenous traditional spaces, provides a culturally embedded and bottom-up pathway for mental health promotion of older adults in China’s age-friendly community construction, and offers diverse experiences for other aging countries worldwide to explore localized and community-based mental health intervention strategies for the older adult.

## 2 Materials and methods

### 2.1 Study setting and design

The Dongmao Street Teahouse was founded by Jian Ming, a master’s graduate in philosophy with deep roots in Huxiang culture and a track record of involvement in projects such as 7up and Super Wenheyou. After establishing the brand “Yunmeng Shanhai” in 2021, he initiated the teahouse project within the former site of the Changsha Second Light Industry Collective Auditorium, originally constructed in 1952. Prior to its opening in September 2024, the development team conducted extensive research into the Changsha Local Chronicles, aiming to authentically reconstruct the city’s traditional public life and culinary memory. By renovating the old industrial structure in a manner that preserved its original character, and by introducing a community-oriented pricing strategy—featuring affordable staples such as scallion oil cakes, sunflower seeds, and tea—the teahouse quickly attracted local older adult residents, who became a daily presence and formed the foundation of its community life.

It has cultivated deep and multifaceted interactions with the older adult population, positioning them as the cornerstone of its community model. Constituting over 40% of its patrons, with some staying from 9 a.m. to 5 p.m. daily, these people over 65 are not merely customers but integral to the space’s authentic character. The teahouse facilitates this through an “older adult-friendly” design, featuring accessible pricing, age-appropriate furniture, and a pressure-free environment where they can linger for hours. This interaction serves a profound social function: it addresses the critical need for social connection among a rapidly aging population, transforming the teahouse into a “third space” that fills the void left by shrinking family structures. Here, older adult patrons form spontaneous social circles, engage in hobbies like sketching, and participate in cultural activities such as watching resident retired artists’ troupes perform Hunan opera, reinforcing their cultural identity. Simultaneously, their daily presence creates an authentic, living tableau of local life—a “human tapestry”—that naturally attracts younger generations and tourists, fostering spontaneous intergenerational dialogue and transforming the teahouse into a vibrant “urban cultural living room” where collective memory is both preserved and shared. It is suitable for exploring the dynamic construction process of emotional community through a qualitative grounded theory design (27).

### 2.2 Data collection

Data were collected between March and September, 2025. Consistent with grounded theory’s cyclical logic, data collection and analysis proceeded simultaneously. We coded the incoming data, and adjusted subsequent data collection to follow up on emerging themes, until we reached theoretical saturation. Our data comes from two core sources: participatory observation at Dongmao Street Teahouse, and in-depth interviews with its older adult regulars.

First, participatory observation. Members of the research team visited Dongmao Street Teahouse 2–3 times per week as regular customers, with each visit lasting 2–4 h, covering morning, midday, and afternoon operating hours. We documented the teahouse’s daily operations, participants’ spatial use habits, interaction patterns, unwritten group norms, and the overall atmosphere of the space. After each visit, we completed detailed field notes within 24 h, including objective observations, researcher reflective memos, and preliminary conceptual insights.

Second, in-depth interviews. Interviewees were selected using theoretical sampling, with inclusion criteria: (1) aged 65 or older; (2) regular visitors to Dongmao Street Teahouse, defined as visiting at least 3–5 times per week; (3) able to communicate clearly and willing to participate in the interview. We ensured sample heterogeneity by balancing gender, age, former occupation, and residential neighborhood. Interviews were conducted in quiet corners of Dongmao Street Teahouse to avoid disruption. Our semi-structured interview guide focused on participants’ motivations for visiting, daily activities in the teahouse, social interaction experiences, emotional attachment to the space, mental health benefits, and views on the teahouse’s future. We adjusted the guide dynamically as new themes emerged from coding. All interviews were audio-recorded with verbal informed consent, and transcribed verbatim within 24 h. We interviewed 25 older adults total: 22 transcripts were used for formal coding analysis, and 3 were reserved for theoretical saturation testing. Participant characteristics are summarized in Table 1.

**Table 1:** Demographic characteristics of participants (*N* = 25).

ID	Gender	Age	Former occupation	Resident in Fengquan Gujing community?
DMJ-INT-01-250829	Male	77	Engineer	No
DMJ-INT-02-250829	Male	71	Teacher	No
DMJ-INT-03-250829	Male	69	Self-employed	Yes
DMJ-INT-04-250829	Male	66	Private enterprise employee	No
DMJ-INT-05-250829	Male	67	Soldier	No
DMJ-INT-06-250829	Male	66	State-owned enterprise employee	No
DMJ-INT-07-250829	Female	67	Freelancer	No
DMJ-INT-08-250829	Male	71	State-owned enterprise employee	No
DMJ-INT-09-250829	Male	70	Engineer	No
DMJ-INT-10-250829	Male	68	Teacher	Yes
DMJ-INT-01-250903	Male	78	Freelancer	Yes
DMJ-INT-02-250903	Female	67	Private enterprise employee	No
DMJ-INT-03-250903	Male	75	State-owned enterprise employee	Yes
DMJ-INT-04-250903	Male	79	Teacher	No
DMJ-INT-05-250903	Female	73	Teacher	No
DMJ-INT-06-250903	Male	68	Freelancer	No
DMJ-INT-07-250903	Male	68	Public institution employee	No
DMJ-INT-08-250903	Male	73	Private enterprise employee	No
DMJ-INT-09-250903	Male	71	Private enterprise employee	No
DMJ-INT-10-250903	Male	67	Private enterprise employee	No
DMJ-INT-11-250903	Male	75	Public institution employee	No
DMJ-INT-12-250903	Male	69	Civil servant	Yes
DMJ-INT-13-250903	Male	72	Private enterprise employee	No
DMJ-INT-14-250903	Female	66	Public institution employee	No
DMJ-INT-15-250903	Male	76	Public institution employee	No

It should be noted that the interviewees of this study are older adult regulars with high-frequency visits to the teahouse. The generation of the research conclusions is highly correlated with the characteristics, needs, and spatial use behaviors of this group, and the generalizability of the findings needs to be further verified among older adult groups with different characteristics and in diverse types of scenarios.

### 2.3 Data analysis

We followed Strauss and Corbin’s three-stage coding process (28): open coding, axial coding, and selective coding. The 25 transcripts were imported into NVivo 15. To ensure coding reliability, two researchers with extensive qualitative research experience completed coding independently, then cross-checked results. Discrepancies were resolved through full team discussion until consensus was reached.

Open Coding: we broke down, compared, conceptualized, and categorized the raw data, line by line, for all 22 interview transcripts and field notes from Dongmao Street Teahouse. We used no pre-set theoretical framework; all initial codes were derived directly from the raw data. Through repeated comparison and refinement, we extracted 18 initial categories (Table 2).

**Table 2:** Examples of open coding: representative quotes and initial categories.

Raw interview examples	Category
It’s lively here, has the flavor of old Changsha. Drinking tea and eating noodles here is very pleasant for me. The environment here, for example, that inscription stele over there, it’s from the old Wuyi Square in Changsha in the 60s. I played there as a child, so it has a very strong old Changsha charm.	Nostalgic aesthetics
I think the environment here is quite good, very flavorful. Also, the tea and stuff here are cost-effective. Overall, it’s good.	Practical function
It’s about happiness and relaxation. In the morning, sitting at a table with a few old brothers, having tea together, just meeting every day is enough. Drinking tea, chatting, talking about daily life, sitting until 10:30 or 11:00, then going back for lunch and rest, playing mahjong in the afternoon. That’s older adult life.	Ritualized daily interaction
One thing to mention is, those who bring dogs should not let the dogs sit on the seats. Dogs are dirty, they soil the seats, how can others sit then? So it depends on personal quality. You cannot ask others not to bring dogs, it’s a business open to all, you can only depend on people’s quality.	Informal rule construction
I’m from this community. I come almost every day, from morning until noon when I go back for lunch.	High-frequency routine participation
What impressed me most is the teahouse’s attitude towards customers since it opened. Initially, customers raised many issues, like teapots being easily broken, poor floor hygiene, inconvenient toilets, etc. The teahouse adopted them one by one and tried its best to solve them. I think this is precious.	Host–guest interaction
Chatting with friends here, talking about how the current overall environment is, business is hard, maybe sometimes complaining a bit. We rarely complain at home.	Interaction with friends/relatives
Usually, people here aren’t very familiar. We meet, sit down, and chat. It does not need to be a very close relationship. It feels like if we hit it off, we just drink tea together and chat about the teahouse or such.	Situational spontaneous interaction
I can chat about anything with anyone. Like, since you study politics, I can talk politics with you, for example, Sino-US relations... Current domestic issues, like healthcare, education, employment, food safety, etc., all these need to be addressed.	Public issue discussion
Discussing views on the changes in Changsha now, sometimes recalling how Wuyi Road was widened in the past, what the old train station was like, chatting about these old things.	Life history narrative sharing
The old Changsha I’m familiar with, I feel that flavor is much less now. But here, I am an old Changsha native. Everyone drinks tea together, chats, reminisces. It feels very flavorful, recalling the memories of our generation.	Local identity
Sometimes movies are shown in the evening, like the old open-air movies. Men, women, old and young sit in front of the white screen, very much like the past.	Cultural nostalgia activities
I’ve painted many pictures in the teahouse. For example, them sitting around the stove, cracking melon seeds, drinking tea, chatting. I paint them vividly. I do not need to hire models; the people in the teahouse are vivid, natural, people from life, right? That’s how I can paint the characters well.	Self-expression activities
For us old folks, just having many people to talk to, being lively, makes us happy. It’s about good mood. Everyone talking together, very lively, makes us feel we are not alone.	Psychological stress relief and role transition
I think the atmosphere here is especially good. Sitting inside often makes me think of the old times, warms my heart.	Nostalgic emotion evocation
Sitting here drinking tea feels just like the old times, very flavorful, reluctant to leave.	Emotional place attachment
I identify with it, like this place very much. For us older adult, retired, besides being at home, we come to the teahouse to have fun. There are many old friends here, very lively, more fun than at home.	Emotional community identity
Hope more people, especially young folks, can truly sit down, not for novelty-seeking, but to genuinely feel the charm of this culture, listen to the stories of us “living histories”. Otherwise, when we are gone, these memories will truly disappear.	Call for cultural inheritance
Many features of the original teahouse are lost. For example, when selling buns, you did not need to take them yourself; the shopkeeper could carry 10 trays by hand, each with four buns. Also, the carrying board required hand strength, could carry 13 bowls of noodles. These are all gone now. We used to play with iron hoops. That was in the 1950s. Many things are hard to restore now.	Authenticity maintenance appeal

Axial Coding: we connected the initial categories from open coding, exploring causal, situational, action-interaction, and consequential relationships between categories, to refine core main categories and sub-categories. Through iterative analysis, we identified six main categories. We defined the core connotation and dimensions of each category, and mapped preliminary logical relationships between them (Table 3).

**Table 3:** Results of axial coding: main categories and constituent elements.

Main category	Description	Constituent initial categories
Nostalgic spatial setting	The physical and cultural environment of the teahouse, combining emotionally resonant historical elements with affordable, comfortable, and accessible amenities.	Nostalgic aesthetics, practical function
Routinized social practices	The habitual, patterned, and rule-governed social behaviors that establish order and predictability within the space.	Ritualized daily exchange, informal rule formation, high-frequency, routine participation
Deep Emotional Interaction	Multi-layered social exchanges ranging from casual chats to profound sharing of personal narratives and societal concerns, facilitating emotional exchange and support.	Host–guest interaction, Kin/Friend interaction, situational interaction, public issue discussion, life history sharing
Cultural identity performance	Acts that affirm and reinforce a shared “old Changsha” identity through participation in traditional activities and personal creative endeavors within the teahouse.	Local identity, cultural nostalgia activities, self-expression activities
Emotional belonging formation	The psychological outcome encompassing stress relief, warm reminiscence, a strong bond to the place, and a conscious identification with the social group.	Psychological relief and role transition, nostalgic emotion evocation, emotional place attachment, emotional community identity
Community sustainability	Concern about the fading of old Changsha culture and expectation for the teahouse to undertake heritage functions. Strong desire for the teahouse to maintain its current (pontificated and nostalgic) style, resisting alienation.	Call for cultural inheritance, authenticity maintenance appeal

**Figure 1:**
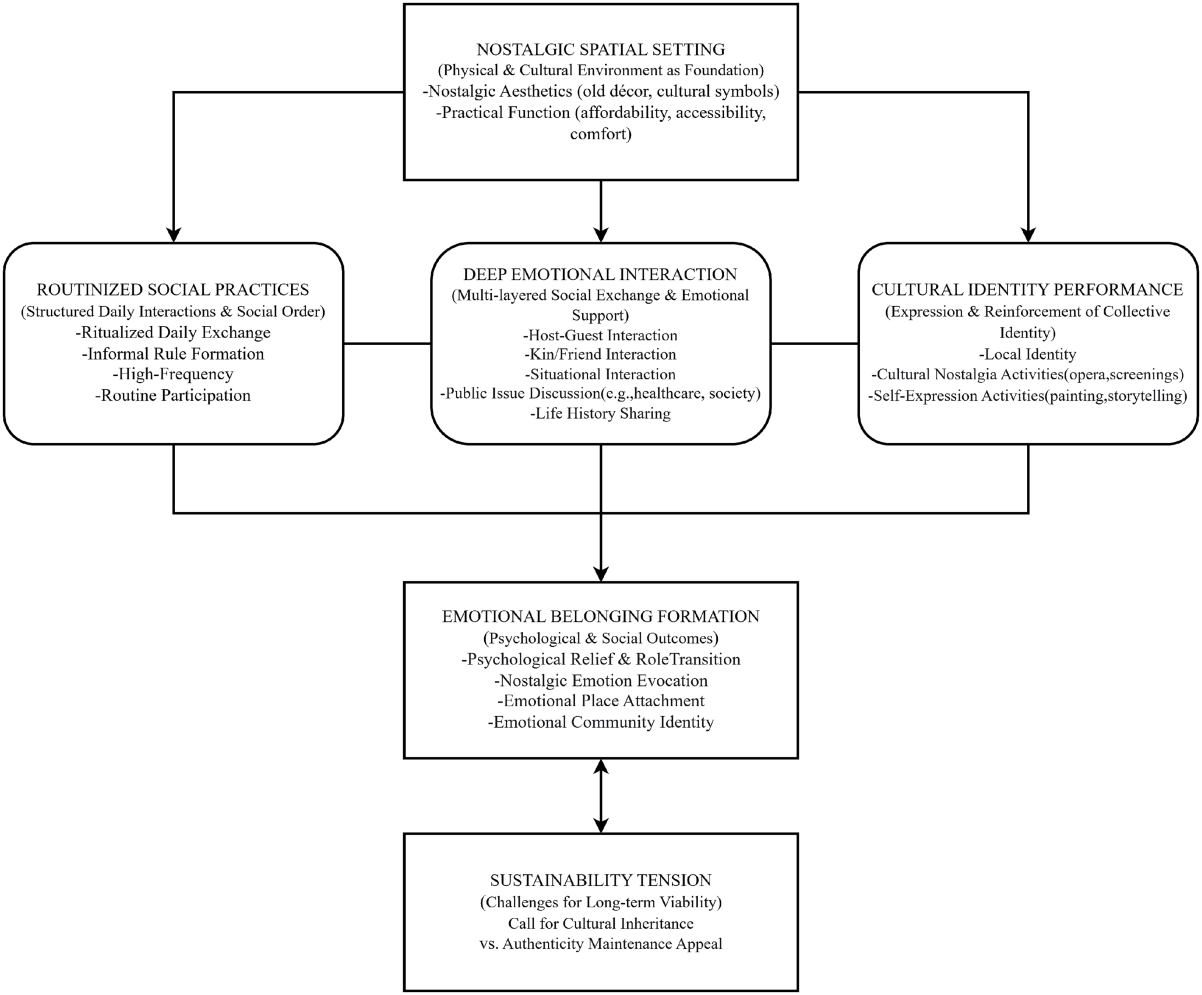
The construction of an older adult emotional community driven by a nostalgic spatial setting and its sustainability tensions.

Selective Coding: We identified the core category from our main categories: The Construction of an Older Adult Emotional Community Driven by a Nostalgic Spatial Setting and Its Sustainability Tensions. Around this core category, we built a cohesive theoretical storyline: precondition → action mechanism → mental health outcome → boundary challenge. We integrated all main and sub-categories into a unified theoretical model (see Figure 1).

Finally, we tested for theoretical saturation. Theoretical saturation is the core criterion for stopping data collection in grounded theory: it is reached when new data produces no new concepts, categories, or relationships between categories. We tested saturation using the three reserved interview transcripts. Coding of these transcripts produced no new concepts, categories, or relationships, confirming that theoretical saturation was achieved.

We confirm that the concepts developed in our three-stage coding process were derived inductively from the empirical data collected at the Dongmao Street Teahouse, rather than being predetermined by existing literature. The coding standards—including dual independent coding, constant comparison, and theoretical saturation—ensured that our findings are grounded in the empirical material rather than imposed by existing theories.

### 2.4 Ethical statement

This study involving human participants was reviewed and approved by the Ethics Review Committee of School of Public Administration, Hunan Normal University, and was conducted in strict accordance with the Declaration of Helsinki. Prior to all data collection activities, all participants were fully informed of the research purpose, data usage rules, their right to withdraw from the study at any time without any negative consequences, and the confidentiality of personal information. Considering the older adult participants’ reading habits and acceptance of formal written documents, verbal informed consent was approved by the ethics committee and obtained from all participants. All audio recordings, interview transcripts and field notes were fully anonymized, with all identifiable personal information (including names, addresses, contact information) removed. The original data are stored in a password-encrypted device of the corresponding author, will not be used for any other purposes beyond this study, and will be destroyed after the publication of the manuscript.

## 3 Results

### 3.1 Overview of the theoretical model

Drawing on the three-stage coding process, this study constructed a dynamic theoretical model of emotional community formation among older adults at the Dongmao Street Teahouse. The model follows a coherent logical sequence: precondition → core mediating processes → mental health outcome → boundary condition. As illustrated in Figure 1, the nostalgic spatial setting serves as the foundational precondition that enables three interrelated core processes—routinized social practices, deep emotional interaction, and cultural identity performance. These processes collectively culminate in the formation of emotional belonging, which represents the key mental health outcome for older adults. However, the sustainability of this emotional community is constrained by an inherent tension between preserving authenticity for existing older adult patrons and adapting to facilitate cultural transmission to younger generations.

### 3.2 The precondition: nostalgic spatial setting

The nostalgic spatial setting emerged from open coding as the initial attractor and essential container for all subsequent social processes. This main category comprises two constituent elements derived directly from participant accounts: nostalgic aesthetics and practical function.

Participants consistently emphasized the emotional resonance of the teahouse’s physical environment. The preservation of old Changsha cultural symbols—such as the inscription stele relocated from the old Wuyi Square—evoked powerful collective memories. As one participant described:

*“The environment here can instantly take me back to my childhood. It has a vibe that modern homes lack. Drinking tea and eating noodles here is very pleasant for me.”* (DMJ-INT-02-250903).

However, the data revealed that nostalgic appeal alone was insufficient; practical functionality was equally critical. Another participant articulated this duality:

*“Good atmosphere is nice, but we come mainly to rest. Good tea, low price, convenient location—that’s what matters. I think the environment here is quite good, very flavorful. Also, the tea and stuff here are cost-effective. Overall, it’s good.”* (DMJ-INT-12-250903).

This combination of emotionally resonant cultural elements with affordable, comfortable, and accessible amenities constitutes the nostalgic spatial setting—the necessary precondition that enables older adults to gather spontaneously and regularly, thereby laying the groundwork for community formation.

### 3.3 Core mediating processes: from spatial setting to emotional connection

Within the nostalgic spatial setting, three interconnected processes unfold, each representing a main category identified through axial coding.

#### 3.3.1 Routinized social practices

The first core process involves the establishment of habitual, patterned social behaviors that create stability and predictability within the teahouse space. This main category comprises four constituent elements: ritualized daily exchange, informal rule formation, high-frequency participation, and routine participation.

Participants described highly regular visitation patterns that structured their daily lives:

*“I come almost every day, from morning until lunch. In the morning, sitting at a table with a few old brothers, having tea together, just meeting every day is enough. Drinking tea, chatting, talking about daily life, sitting until 10:30 or 11:00, then going back for lunch and rest.”* (DMJ-INT-03-250903).

Over time, these repeated interactions generated unwritten social norms that governed behavior within the space. These informal rules emerged organically from collective practice rather than being imposed externally:

*“Seats are first-come, first-served, but you can share a table. We all know our usual spots. One thing to mention is, those who bring dogs should not let the dogs sit on the seats. Dogs are dirty, they soil the seats, how can others sit then? So it depends on personal quality.”* (DMJ-INT-10-250829).

These routinized practices transform the teahouse from a mere physical location into a structured social world where participants share tacit understandings and expectations, creating the stable foundation necessary for deeper emotional connections to develop.

#### 3.3.2 Deep emotional interaction

The second core process encompasses multi-layered social exchanges that range from casual encounters to profound sharing of personal narratives and societal concerns. This main category integrates five constituent elements identified in open coding: host-guest interaction, interaction with friends/relatives, situational spontaneous interaction, public issue discussion, and life history narrative sharing.

The data revealed a spectrum of interaction types, each contributing differently to participants’ emotional lives. Casual, situational interactions provided spontaneous social stimulation:

*“Usually, people here aren’t very familiar. We meet, sit down, and chat. It does not need to be a very close relationship. It feels like if we hit it off, we just drink tea together and chat about the teahouse or such.”* (DMJ-INT-03-250903).

More substantive exchanges occurred through discussions of public affairs, which maintained participants’ sense of civic engagement and intellectual stimulation:

*“I can chat about anything with anyone. Like, since you study politics, I can talk politics with you, for example, Sino-US relations... Current domestic issues, like healthcare, education, employment, food safety, etc., all these need to be addressed.”* (DMJ-INT-01-250903).

The deepest level of interaction involved sharing personal life histories, which served to validate individual experiences and forge collective identity:

*“Discussing views on the changes in Changsha now, sometimes recalling how Wuyi Road was widened in the past, what the old train station was like, chatting about these old things.”* (DMJ-INT-07-250903).

Significantly, participants noted that these emotionally rich interactions were often unavailable elsewhere:

*“Chatting with friends here, talking about how the current overall environment is, business is hard, maybe sometimes complaining a bit. We rarely complain at home.”* (DMJ-INT-02-250903).

The relationship with teahouse staff also contributed to this emotional ecology:

*“What impressed me most is the teahouse’s attitude towards customers since it opened. Initially, customers raised many issues. The teahouse adopted them one by one and tried its best to solve them. I think this is precious. That makes us feel valued.”* (DMJ-INT-03-250903).

These layered interactions collectively generate emotional exchange and mutual support, transforming casual acquaintances into meaningful social bonds.

#### 3.3.3 Cultural identity performance

The third core process involves active affirmation and reinforcement of a shared “old Changsha” identity through participation in culturally meaningful activities. This main category comprises three constituent elements: local identity, cultural nostalgia activities, and self-expression activities.

Participants strongly identified with the teahouse as a space that affirmed their cultural belonging:

*“The old Changsha I’m familiar with, I feel that flavor is much less now. But here, I am an old Changsha native. Everyone drinks tea together, chats, reminisces. It feels very flavorful, recalling the memories of our generation.”*(DMJ-INT-05-250903).

The teahouse facilitated this identity performance through organized cultural activities that evoked collective nostalgia:

*“Sometimes movies are shown in the evening, like the old open-air movies. Men, women, old and young sit in front of the white screen, very much like the past.”* (DMJ-INT-03-250903).

Notably, some participants actively contributed to the cultural life of the space through creative expression, further reinforcing their sense of purpose and identity:

*“I’ve painted many pictures in the teahouse. For example, them sitting around the stove, cracking melon seeds, drinking tea, chatting. I paint them vividly. I do not need to hire models; the people in the teahouse are vivid, natural, people from life, right? That’s how I can paint the characters well.”* (DMJ-INT-02-250903).

This process of cultural identity performance transforms the teahouse from a passive consumption space into an active arena for cultural production and identity affirmation, deepening participants’ emotional investment in the community.

### 3.4 The outcome: emotional belonging formation

The culmination of the three core mediating processes is the development of emotional belonging—a multi-faceted psychological outcome that represents the key mental health benefit for older adults. This main category integrates four constituent elements: psychological stress relief and role transition, nostalgic emotion evocation, emotional place attachment, and emotional community identity.

Participants consistently reported significant psychological benefits from their teahouse participation: *“For us old folks, just having many people to talk to, being lively, makes us happy. It’s about good mood. Everyone talking together, very lively, makes us feel we are not alone.”* (DMJ-INT-07-250903).

The nostalgic atmosphere evoked warm emotions that enhanced psychological well-being: *“I think the atmosphere here is especially good. Sitting inside often makes me think of the old times, warms my heart.”* (DMJ-INT-05-250903).

A profound attachment to the physical space itself was evident in participants’ accounts: *“Sitting here drinking tea feels just like the old times, very flavorful, reluctant to leave.”* (DMJ-INT-08-250829).

Most significantly, participants articulated a conscious identification with the teahouse-based social group, explicitly framing it as a community: *“I identify with it, like this place very much. For us people over 65, retired, besides being at home, we come to the teahouse to have fun. There are many old friends here, very lively, more fun than at home.”* (DMJ-INT-04-250903).

This sense of emotional belonging directly addresses key risk factors for poor mental health in later life—loneliness, perceived isolation, and loss of purpose—by providing sustained companionship, emotional support, and meaningful social identity.

### 3.5 The boundary condition: sustainability tension

While the emotional community generates significant mental health benefits, selective coding revealed an inherent tension that challenges its long-term sustainability. This main category comprises two constituent elements: call for cultural inheritance and authenticity maintenance appeal, which together create a fundamental dilemma.

On one hand, participants strongly desired preservation of the teahouse’s authentic character, resisting changes that might undermine its value:

*“Many features of the original teahouse are lost. For example, when selling buns, you did not need to take them yourself; the shopkeeper could carry 10 trays by hand... These are all gone now. We used to play with iron hoops. That was in the 1950s. Many things are hard to restore now.”* (DMJ-INT-01-250829).

This desire for preservation reflects participants’ recognition that the teahouse’s therapeutic value depends on maintaining the nostalgic spatial setting and informal social environment that enabled community formation.

On the other hand, participants expressed concern about the cultural continuity of their community and a desire to transmit their memories and identity to younger generations:

*“Hope more people, especially young folks, can truly sit down, not for novelty-seeking, but to genuinely feel the charm of this culture, listen to the stories of us ‘living histories’. Otherwise, when we are gone, these memories will truly disappear.”* (DMJ-INT-04-250903).

This call for cultural inheritance reflects participants’ need for a sense of generational continuity and meaning that extends beyond their immediate peer group.

The tension between these two imperatives—preservation of authenticity versus adaptation for intergenerational transmission—constitutes a critical boundary condition for the emotional community’s sustainability. If the teahouse over-adapts to attract younger customers by compromising its authentic character, it risks losing the core attributes that sustain older adult participation. Conversely, if it rigidly preserves its current form without facilitating intergenerational connection, it may fail to meet older adults’ needs for cultural transmission and meaning-making, potentially diminishing their sense of purpose and long-term commitment to the space.

## 4 Discussion

### 4.1 Principal findings and public health implications

Based on the three-stage coding analysis of Grounded Theory, this study constructed a four-stage dynamic model of emotional communities among older adults, which follows the logic of “nostalgic spatial setting as the precondition, three core social–emotional processes as the mediators, formation of emotional belonging as the mental health outcome, and sustainability tension as the boundary condition.” The model systematically addresses the three core research questions raised in the introduction, and clearly elaborates how teahouses, as Third Places deeply embedded in Chinese indigenous culture, nurture emotional communities that serve as core resources for the psychological and social well-being of urban older adults through a bottom-up interaction process. The findings of this study not only resonate with international public health research on the social determinants of health among older adults, but also make important breakthroughs in the interpretation of indigenous mechanisms and the expansion of theoretical boundaries (29, 30).

First, the teahouse functions as a low-threshold health-promoting environment. Its affordability and accessibility address practical barriers to social participation faced by older adults. By providing a setting for routinized, informal contact, it helps maintain social connectivity, a known protective factor against cognitive decline, depression, and mortality (31, 32). Second, the emotional community provides multi-dimensional social support. The deep interactions—especially life-story sharing—facilitate emotional support and validate personal identity and worth post-retirement. Discussions of public issues provide informational support and maintain a sense of civic engagement. The collective identity offers appraisal support, buffering against the negative impacts of ageism. Third, the formation of emotional belonging directly targets key risk factors for poor mental health in later life, namely loneliness, perceived isolation, and lack of purpose (33, 34). The teahouse community offers a sense of purpose through cultural stewardship, and consistent companionship.

Extending the point above regarding low-threshold environments, our analysis further illuminates how the material and sensory attributes of the teahouse.

#### 4.1.1 Theoretical contributions and dialogue with existing literature

Our findings advance existing research in three key ways.

First, this study extends the Third Place Theory to the non-Western collectivist context and clarifies the complete mechanism through which indigenous Third Places promote the mental health of older adults. Most existing studies on Third Places are rooted in Western individualistic contexts, focus on the neutrality and social functions of Third Places, and have verified their value in providing social opportunities for older adults (9, 10). However, based on the empirical results from grounded coding, this study finds that in the Chinese collectivist context, the core attractiveness and driving mechanism of Dongmao Street Teahouse as a Third Place lie in its nostalgic spatial setting that evokes the collective memory of older adults. This core category is derived from indigenous concepts such as “nostalgic aesthetics” and “cultural nostalgia activities” extracted through open coding, which is distinct from the core driving logic of Western Third Places. Meanwhile, unlike Western Third Places that often facilitate superficial and random social interactions, the empirical results of this study demonstrate that through the chain mediating process of “routinized social practices - deep emotional interaction–cultural identity performance,” the teahouse eventually evolves into an emotional community with deep mutual assistance relationships, shared collective identity and a strong sense of emotional belonging, rather than a mere social venue. This finding not only breaks through the cultural context limitations of Western Third Place Theory and expands its theoretical connotation in non-Western collectivist cultures, but also clarifies the complete dynamic action path from physical space to mental health based on empirical data: nostalgic spatial setting → routinized social interaction practices → multi-dimensional emotional connection and identity construction → formation of emotional belonging → improvement of mental health and well-being. It fills the key gap in existing research, which mostly focuses on correlation analysis rather than in-depth exploration of the underlying causal mechanism.

Second, we enrich the theory of emotional community. Existing research on emotional community has focused largely on residential neighborhoods or online communities, with a heavy emphasis on top-down community building (23, 24). Our study reveals the bottom-up formation of an emotional community among older adults in an informal commercial space. We identify the core roles of nostalgic space, routine practice, emotional interaction, and identity performance in this process, expanding the application of emotional community theory in aging research.

Third, we add to the literature on locally rooted active aging. Most existing research on active aging in China focuses on formal older adult care systems, framing older adults as passive recipients of services. Our study confirms that grassroots, informal local spaces like Dongmao Street Teahouse are a critical complement to formal care (14, 15), and that older adults are active architects of their own spaces and communities. We offer a culturally embedded, low-threshold, non-clinical pathway for mental health promotion in later life, enriching the local application of active aging theory (4–6).

Building on the aforementioned theoretical contributions, this study further clarifies the universal core elements transferable across contexts and the context-specific elements rooted in Chinese local settings within the teahouse model.

Among them, the transferable core elements include four dimensions: first, the principle of low-threshold and accessible space provision to address the economic and physical barriers to social participation among older adults; second, the creation of informal social scenarios that respect the autonomous leadership of older adults, rather than top-down designed structured activities; third, the community growth logic of nurturing in-depth emotional bonds and a sense of community belonging through routinized daily interactions; fourth, the action pathway of leveraging the cultural attributes of space to evoke collective memory, strengthen identity, and thereby promote mental health.

The context-specific local elements include: the spatial form with traditional Chinese tea culture as the carrier, the construction of nostalgic settings centered on regional collective memory, and the interaction mode of emotional communities based on Chinese collectivist culture. These elements bear distinct local cultural characteristics and cannot be directly replicated; they need to be adapted in line with the cultural traditions and indigenous spatial forms of different countries and regions. This delineation provides a clear reference framework for active aging practices in the context of global multiculturalism.

#### 4.1.2 Comparative analysis of third places in Chinese and Western contexts and homologous local older adult care spaces

To further clarify the unique value of traditional urban teahouses as third places for older adults and respond to academic discussions on the morphological differentiation of third places against different cultural backgrounds, this study conducts a comparative analysis from two dimensions: the essential differences between third places in Chinese and Western contexts, and the characteristic distinctions between teahouses and local formal older adult care spaces. It highlights the core advantages of teahouses: low threshold, unstructured, and self-governable and provides targeted references for the planning and construction of age-friendly spaces.

In terms of the essential differences between Third Places in Chinese and Western contexts, although both Western Third Places and traditional Chinese teahouses fulfill the functions of alleviating social isolation and facilitating informal social interaction, there are empirically verifiable significant differences between the two in cultural core, operational logic, interaction mode and ultimate value output based on the empirical findings of this study. Western Third Places, such as European cafes and American community centers, are deeply influenced by individualistic cultural norms (9, 10): European cafes are centered on commercial operation, and their value for older adults is mainly reflected in providing random social opportunities. The participation of older adults is characterized by strong voluntariness and mobility, making it difficult to form stable communities with emotional bonds. American community centers focus on structured and professionalized activities, where the social interactions of older adults must be carried out within a pre-designed activity framework, representing a top-down designed service provision.

In contrast, Dongmao Street Teahouse in Changsha in this study is rooted in Chinese collectivist values and regional cultural traditions, with its commercial attribute significantly weakened and its social and cultural attributes occupying a dominant position. As evidenced by the coding results, the teahouse takes the nostalgic spatial setting as the core link, and realizes the long-term, high-frequency and stable spontaneous gathering of older adults by evoking collective memory and cultural identity. The content, rhythm and norms of social interaction within the space are completely independently dominated by older adult users rather than designed by the operator, eventually forming an emotional community centered on in-depth emotional bonds, mutual support and shared identity. The essential difference between the two is that the core value of Western Third Places for older adults is “providing social opportunities,” while the core value of Chinese indigenous teahouses for older adults is “nurturing emotional communities”—this is precisely the unique localized form of Third Places in the context of collectivist culture.

In terms of the characteristic distinctions between teahouses and homologous local formal older adult care spaces, compared with formal older adult-oriented spaces constructed in a top-down manner by the government, such as community activity centers and universities for the older adult, the teahouse in this study has three unique, empirically verifiable advantages in meeting the daily social and mental health needs of older adults, as validated by the study’s empirical results: low threshold, unstructured nature, and autonomous governance.

Community activity centers and universities for the older adult are defined by the core characteristics of structured activities and standardized management (14, 15). The content of activities, participation time, and interaction rules are all pre-set by staff, strictly confining the social behaviors of older adults within the pre-defined activity framework. This makes it difficult for older adults to express their social needs in an autonomous and personalized manner, and their participation is mostly a one-way, passive acceptance of services, which hinders the development of sustained, stable emotional bonds and a sense of community belonging.

Conversely, the coding results of this study demonstrate that the core advantages of the teahouse fully align with the essential needs of older adults. First, the low-threshold attribute, which is reflected in both the economic dimension—the extremely low cost of tea consumption imposes no financial burden on older adults—and the participation dimension. The open space has no entry barriers for participation: no registration or appointment is required, and older adults can enter and exit at any time and sit freely, which fits the daily travel habits of older adults and serves as the foundation for the formation of “high-frequency routine participation”. Second, the unstructured attribute. There is no fixed activity procedure or mandatory interaction requirement within the space, and older adults can freely choose any activity such as sitting quietly, chatting, tea tasting, and creative work, which fully adapts to their energy levels and social willingness. This is the core prerequisite for the emergence of “ritualized daily interaction and in-depth emotional exchange”. Third, the attribute of autonomous governance. The social order and informal interaction norms within the teahouse are spontaneously formed and jointly observed by older adult regulars through long-term daily interactions. The operator only coordinates to optimize basic services without interfering in the social process, which enables older adults to become the true masters of the space, and in turn fosters a strong sense of ownership, place attachment, and community belonging (18, 24).

These unique advantages enable the teahouse to become an organically evolved social space independently led by older adults. It forms a strong functional complementarity with formal older adult care spaces, and together they constitute the spatial support system for age-friendly communities.

### 4.2 Sustainability tension: core challenges to the survival of emotional communities and their impacts on mental health

Through selective coding, this study identifies “sustainability tension” as the core boundary condition of the dynamic model of emotional communities. The core of this tension lies in the inherent contradiction between older adult regulars’ demand for the preservation of the teahouse’s authenticity and their expectation for its intergenerational cultural transmission. This tension is not an additional issue irrelevant to mental health, but a key factor that directly affects the long-term survival of emotional communities, and in turn, the sense of emotional belonging and mental well-being of older adults. Its mechanism of action and impact pathways, all derived from the interview and coding results of this study, are mainly reflected in two aspects.

First, the authenticity of the teahouse space is the core foundation for the construction of the nostalgic setting, as well as the precondition for the formation of the emotional community. Once compromised, it will directly erode the mental health benefits of older adults. The coding results of this study show that older adults’ core identification with the teahouse first stems from its nostalgic spatial attribute of “the charm of old Changsha.” This authenticity, which carries collective memory, is the primary prerequisite for attracting high-frequency visits from older adults and enabling the formation of routinized social practices. If the teahouse undergoes excessive commercial and internet-fame-oriented renovation to attract young customers, which undermines the original nostalgic spatial atmosphere, low-threshold consumption model, and open and inclusive social environment, it will directly disintegrate older adults’ place attachment and identity with the space. This will in turn lead to the breakdown of stable, routinized social practices, the gradual dissolution of the established emotional community, and ultimately the loss of this vital mental health support setting for older adults. As one older adult interviewee stated, “We hope it will always stay the way it is, not become flashy and gimmicky. Otherwise, we old folks will have nowhere to go.” The preservation of the spatial authenticity is directly linked to older adults’ sense of belonging, sense of security, and sustained mental health benefits.

Second, the lack of intergenerational transmission impairs older adults’ acquisition of a sense of meaning, which is identified in this study as a critical component of mental health promotion. The results of this study indicate that the formation of emotional belonging stems not only from the companionship and emotional support of peer groups, but also from the sense of value and meaning that older adults gain through sharing and transmitting the culture of “old Changsha.” This is an important pathway to counteract the sense of role loss after retirement and enhance self-identity. Older adult interviewees generally expressed the appeal that “We hope young people will come to listen to our stories, otherwise these old memories will disappear with us.” This expectation for cultural transmission is a key source for older adults to gain self-worth identity and buffer the sense of role loss after retirement. If the teahouse rigidly adheres to its original model and fails to facilitate intergenerational cultural connection and interaction, older adults’ demand for cultural transmission will go unmet, which will in turn undermine their sense of self-worth and weaken the mental health promotion effect of the space.

This inherent tension is also a common challenge faced by indigenous Third Places globally. Western community cafes and social spaces for older adults often grapple with the contradiction between modernization renovations to attract young customers and the risk of losing their core older adult patrons. For traditional Chinese teahouses, however, balancing this tension is even more challenging, as the authenticity of the space is not merely a matter of environmental style, but the core foundation for sustaining the emotional community. Therefore, public health and community development policies must address this issue prudently. Rather than simply equating the commercial renovation of teahouses with “revitalization,” policies should support such spaces to achieve inclusive, moderate intergenerational connection and sustainable development on the premise of safeguarding the core needs of older adult users and preserving the essence of the space’s authenticity, so as to ensure the long-term viability of these mental health support settings for older adults.

### 4.3 Limitations and future research

This study has the following four limitations, and we propose corresponding future research directions addressing these limitations.

First of all, selection bias and survivorship bias in the study sample may affect the scope of application of the research conclusions. All interviewees in this study are older adult regulars with high-frequency visits to Dongmao Street Teahouse (3–5 times or more per week), and most of them are urban older adults who held stable occupations prior to retirement, had favorable economic conditions, and possessed full independent mobility. This sampling strategy aligns with the theoretical sampling principle of “information richness” in Grounded Theory, enabling us to conduct an in-depth exploration of the complete construction process and inherent mechanisms of emotional communities. However, it inevitably introduces selection bias and survivorship bias—the research conclusions cannot cover older adults with mobility impairment, financial burdens, heavy family caregiving responsibilities, or an aversion to such public social spaces. Such groups are precisely the high-risk populations for social isolation and mental health problems, yet they are unable to access teahouse spaces of this kind and obtain corresponding health benefits. Therefore, the scope of application of the dynamic model of emotional communities constructed in this study is mainly limited to urban retired older adults with independent mobility, social willingness, and basic economic capacity. The model has certain limitations in terms of inclusiveness and generalizability, and future research needs to include older adult groups with more diverse characteristics to verify the applicability of the model across different populations.

Second, the limitation of a single case and regional culture undermines the generalizability of the theoretical model. This study adopts a single-case Grounded Theory design, with the field site limited to Dongmao Street Teahouse in Changsha, which is deeply embedded in Huxiang culture. Its spatial characteristics, cultural connotations, and interaction patterns of older adults all bear distinct regional cultural features. The validity and generalizability of the theoretical model constructed in this study need to be tested and refined in scenarios of different regional cultures (e.g., community activity rooms in northern China, old teahouses in the Sichuan-Chongqing region, herbal tea shops in the Guangdong-Hong Kong-Macao region) and different types of indigenous Third Places (e.g., urban parks, community fairs, universities for the older adult). Future research can adopt a multiple-case comparative design to compare differences in the construction mechanisms of emotional communities for older adults in indigenous Third Places of different regions and types, verify and optimize the theoretical model of this study, and explore moderating factors under different socio-economic and cultural backgrounds.

Third, the singularity of the research perspective makes it difficult to fully cover the multi-dimensional impacts of spatial governance. This study mainly adopts the internal perspective of older adult users, and the collected data are mainly derived from interviews and participant observation with older adults. It does not incorporate the perspectives of other stakeholders, including teahouse operators, community managers, young consumers, and surrounding community residents. This makes it difficult to comprehensively and completely understand the governance dynamics of such spaces, the influencing factors for their sustainable development, and the differences in demands for the space across different groups. Future research can incorporate a multi-stakeholder perspective, and through interviews and surveys with multiple subjects, gain a more comprehensive understanding of the operational logic, development dilemmas, and policy support needs of such indigenous Third Places, so as to provide a more holistic evidence base for policy formulation.

Fourth, as a qualitative study, the theoretical model constructed in this study has not been verified by large-sample quantitative analysis. This study constructed a dynamic formation model of emotional communities through Grounded Theory and extracted relevant core categories, but these qualitative constructs have not yet been converted into measurable variables, and the action pathways of the model have not been quantitatively tested with a large sample. Two highly promising future research directions are identified: first, to develop measurement scales for core constructs such as “perceived spatial nostalgia,” “depth of emotional interaction,” and “sense of community belonging,” and improve relevant measurement tools; second, to test the action pathways constructed in this study through large-sample questionnaire surveys, conduct a quantitative analysis of the effects of teahouses as Third Places on older adults’ loneliness, life satisfaction, and mental health status, verify the health benefits of such communities on a larger scale, and provide more solid empirical evidence for policy promotion.

### 4.4 Policy and practical implications

Based on the core findings of this study, combined with the aforementioned delineation of transferable core elements and context-specific local elements, this study provides actionable and targeted implications for the construction of age-friendly cities in China, as well as for public health policies on active aging in the context of global multiculturalism.

#### 4.4.1 Indigenous implications for domestic age-friendly community construction

First, urban planning and public health policies should explicitly incorporate “age-friendly Third Places” as a core component of the construction of age-friendly cities and communities. On the one hand, it is necessary to systematically identify, preserve and revitalize existing indigenous informal public spaces such as teahouses that are widely popular among older adults in urban areas. Targeted policy support, including rent reductions, tax incentives, and utility subsidies, should be provided to space operators that have long served older adults, maintained low-threshold consumption, and preserved local cultural characteristics, so as to reduce their operational pressure and safeguard these mental health support settings for older adults. On the other hand, in the process of urban renewal and the planning of new communities, open informal public spaces adapted to the needs of older adults should be reserved in advance. The design of such spaces should integrate both local nostalgic cultural elements and barrier-free, age-friendly hardware requirements, to provide spatial carriers for older adults to independently form social communities.

Second, community social work services should be embedded in such indigenous Third Places to act as “facilitators of social connection.” Community social workers should proactively identify informal spaces where older adults in the jurisdiction gather spontaneously, rather than limiting their work to formal venues such as community activity centers. By organizing lightweight activities such as “Urban Memory Sharing Sessions” and “Oral History of Local Culture,” they can promote intergenerational dialogue and cultural inheritance, and consciously build social bridges. While meeting older adults’ needs for emotional connection and value realization, these activities can also attract young people to learn about local culture, thus alleviating the inherent tension in the sustainability of teahouse spaces.

Besides, for space operators, a sustainable development strategy of “preserving the core while innovating at the periphery” should be adopted. “Preserving the core” means adhering to the three fundamental pillars that safeguard the core needs of older adults: preserving the nostalgic cultural atmosphere and authenticity of the space, maintaining a low-threshold consumption model, and protecting an open and inclusive social environment autonomously led by older adults — these constitute the core value of the space. “Innovating at the periphery” means that on the premise of not undermining the core pillars, lightweight, experiential new elements such as tea culture workshops, mini-exhibitions of local history, and local intangible cultural heritage experience activities can be introduced to attract a more diverse customer base. This will realize intergenerational inclusiveness and long-term sustainable development of the space, while avoiding the crowding-out effect on core older adult users caused by excessive commercial renovation.

#### 4.4.2 Transferable implications for global active aging practices

The core logic identified in this study also has important reference significance for other countries and regions facing the challenge of population aging around the world. The core transferable implications include three points:

First, public health policies and urban planning should move beyond the inherent mindset that “the mental health of older adults can only be addressed through the formal older adult care service system,” and attach importance to the core value of informal, indigenous Third Places in promoting the mental health of older adults. Compared with clinical mental health interventions and structured older adult care services, such Third Places spontaneously used by older adults have the advantages of low threshold, stigma-free access and strong sustainability. They serve as a critical supplement to the formal older adult care service system, and should be incorporated into the policy framework of public health and urban planning with corresponding recognition and support.

Second, the construction of age-friendly spaces should adhere to the “older adults’ needs-centered” principle, respect the autonomous leadership of older adults, rather than designing standardized services and activities in a top-down manner. This study confirms that older adults are active architects of their own social spaces and communities. The core of policy and planning is not to “pre-design” everything for older adults, but to provide accessible, inclusive and open spatial carriers, allowing older adults to independently form routinized social interactions and spontaneously cultivate communities with emotional support functions. This core logic is applicable to different cultural and social contexts.

Third, intervention strategies for active aging should be fully integrated with local cultural traditions, and explore spatial carriers and social modes rooted in local culture. Countries and regions have different cultural traditions, and the corresponding forms of indigenous Third Places also vary. Policy making should not directly replicate the spatial models of other countries. Instead, based on local cultural traditions, it should identify indigenous space types that local older adults gather in spontaneously and have cultural resonance with, provide targeted support, and build mental health support settings that fit the local cultural context and truly meet the needs of older adults.

## 5 Conclusion

Taking Dongmao Street Teahouse in Changsha as a typical case, this study adopts a qualitative research approach grounded in Grounded Theory to explore the dynamic process, inherent mechanisms, and mental health promotion effects of traditional urban teahouses in China — as Third Places — in nurturing emotional communities for older adults, and systematically addresses the three core research questions raised in the introduction.

The core findings of this study can be summarized into three points:

For a start, within the context of Chinese cities, the construction of emotional communities for older adults in teahouses is a four-stage dynamic process. It takes the nostalgic spatial setting as the precondition, and ultimately forms a sense of emotional belonging through three interrelated core processes: routinized social practices, deep emotional interaction, and cultural identity performance. Meanwhile, the sustainability tension between the preservation of authenticity and intergenerational cultural transmission serves as the core boundary condition for the survival of this emotional community.

Next, the teahouse-based emotional community has a significant positive effect on the mental health of urban older adults, which is realized through three main pathways: first, alleviating older adults’ loneliness and sense of social isolation by maintaining stable social connectivity; second, relieving psychological stress and facilitating role transition and adaptation after retirement through in-depth emotional interaction and peer support; third, enhancing older adults’ sense of self-worth and meaning through the confirmation and transmission of cultural identity, which ultimately fosters a strong sense of place attachment and community belonging, and improves overall psychological well-being.

Lastly, the teahouse-driven model for promoting older adults’ mental health contains both cross-culturally transferable universal core logic and context-specific elements rooted in Chinese indigenous culture. Its core experience can provide a clear reference framework for public health policies on active aging in the context of global multiculturalism.

This study confirms that informal Third Places such as traditional urban teahouses — which are deeply embedded in local culture and spontaneously used by older adults — can serve as powerful community health assets for promoting the well-being of older adults. These spaces drive the formation of emotional communities through a bottom-up organic growth process, and the meaningful social participation, multi-dimensional social support, reinforced cultural identity, and deep sense of belonging provided by such emotional communities are exactly the core protective factors for mental health in later life. It should be clarified that the conclusions of this study are mainly applicable to urban retired older adults with independent mobility and social willingness, and the generalizability of the findings needs to be further verified in populations with different characteristics and in scenarios with diverse regional and cultural backgrounds.

For public health practice and policy-making amid global population aging, the core implications of this study are as follows: First, informal indigenous spaces where older adults gather spontaneously should be identified, protected, and supported as important community health assets, rather than only focusing on top-down constructed formal older adult care service facilities. Second, the core design principles of Third Places — accessibility, inclusiveness, and neutrality — should be fully integrated into the planning and construction of age-friendly communities and public spaces, with respect for the autonomous leadership of older adults, to provide suitable spatial carriers for their informal social interactions. Third, mental health intervention strategies for active aging should be fully rooted in local cultural traditions, explore spatial and social modes that are familiar and recognized by local older adults, and build health support scenarios that combine cultural adaptability and sustainability. Fostering such bottom-up communities that can provide sustained emotional support is a core pathway to addressing the challenges of population aging, advancing the goals of active aging, and improving the mental health of older adults.

## Data Availability

The original contributions presented in the study are included in the article/supplementary material, further inquiries can be directed to the corresponding author/s.
